# Three dimensional electron microscopy reveals changing axonal and myelin morphology along normal and partially injured optic nerves

**DOI:** 10.1038/s41598-018-22361-2

**Published:** 2018-03-05

**Authors:** Marcus K. Giacci, Carole A. Bartlett, Minh Huynh, Matt R. Kilburn, Sarah A. Dunlop, Melinda Fitzgerald

**Affiliations:** 1 Experimental and Regenerative Neurosciences, School of Biological Sciences, The University of Western Australia, 35 Stirling Hwy, Perth, 6009 Western Australia Australia; 2 Centre for Microscopy, Characterisation, and Analysis, The University of Western Australia, 35 Stirling Hwy, Perth, 6009 Western Australia Australia; 3 Australian Centre for Microscopy and Microanalysis, The University of Sydney, City Road, Sydney, 2006 New South Wales Australia; 4 Curtin Health Innovation Research Institute, Curtin University, Bentley, 6102 Western Australia Australia; 5 Perron Institute for Neurological and Translational Science, Sarich Neuroscience Research Institute, 8 Verdun St, Nedlands, 6009 Western Australia Australia

## Abstract

Following injury to the central nervous system, axons and myelin distinct from the initial injury site undergo changes associated with compromised function. Quantifying such changes is important to understanding the pathophysiology of neurotrauma; however, most studies to date used 2 dimensional (D) electron microscopy to analyse single sections, thereby failing to capture changes along individual axons. We used serial block face scanning electron microscopy (SBF SEM) to undertake 3D reconstruction of axons and myelin, analysing optic nerves from normal uninjured female rats and following partial optic nerve transection. Measures of axon and myelin dimensions were generated by examining 2D images at 5 µm intervals along the 100 µm segments. In both normal and injured animals, changes in axonal diameter, myelin thickness, fiber diameter, G-ratio and percentage myelin decompaction were apparent along the lengths of axons to varying degrees. The range of values for axon diameter along individual reconstructed axons in 3D was similar to the range from 2D datasets, encompassing reported variation in axonal diameter attributed to retinal ganglion cell diversity. 3D electron microscopy analyses have provided the means to demonstrate substantial variability in ultrastructure along the length of individual axons and to improve understanding of the pathophysiology of neurotrauma.

## Introduction

Injury to white matter tracts of the central nervous system (CNS), such as the optic nerve, results in a complex metabolic, cellular and structural response. Initially intact neurons and glia surrounding the site of insult are vulnerable to a disruptive set of cellular and molecular cascades known as secondary degeneration, leading to further loss of function^1–3^. Secondary degeneration can be investigated using an established model involving partial transection of the optic nerve^1,4^. After injury to the dorsal aspect of the nerve, retinal ganglion cell (RGC) axons in ventral nerve adjacent to the primary injury site remain intact and spatially separated from the primary injury, but are vulnerable to secondary degeneration. Using single transverse sections through the nerve at the injury site, immunohistochemical and ultrastructural analyses of surviving ventral optic nerve tissue undergoing secondary degeneration reveal a 20% loss of initially spared axonal profiles 2 weeks after injury^1,5^, followed by alterations in axon diameter and myelin integrity in remaining axons at 3 months. Therapeutic strategies that limit damage during secondary degeneration are critical for preserving long term functional outcomes following neurotrauma^6^ and the partial optic nerve transection model has proved useful for assessing the efficacy of therapeutic interventions for preserving axons and myelin^5,7–9^. However, to date, ours and other studies of white matter trauma and potential treatments have been confined to two-dimensional (2D) analyses of single sections to quantify ultrastructural changes in axons and their myelin following injury^10–13^. Such analyses may miss subtleties of pathology and changes in structure along the length of individual axons and their ensheathing myelin internodes and therefore confound interpretation.

Axon diameter^14^, along with myelin thickness^15^, internode length^16^ and paranode gap^17^ determine the functional properties of nerves. There is a strong link between structure and function in the CNS and, as such, the characterisation of ultrastructural properties has proved useful in exploring the pathology of neurological conditions. Specifically, axon diameter has been used to determine conduction velocity along various pathways^18^. These measures rest upon the commonly accepted concept that an axon’s shape does not substantially vary over its length^19^. As such, single section measurements often used when quantifying axon and myelin morphology give little consideration to the possibility that there may be varying morphological or pathological changes along the length of individual axons^20^. Serial block face-scanning electron microscopy (SBF SEM) with supervoxel-based segmentation enables examination of subtle changes in axonal diameter, fiber diameter, myelin thickness and decompaction along a length of myelinated axon. The current study characterises morphological changes to spared RGC axons and their associated myelin sheaths in optic nerves from normal animals and following partial optic nerve transection. Intra-axonal variability of RGC axon diameter, fiber diameter, myelin thickness, G-ratio, decompaction, paranodal gaps and mitochondrial length and number are quantified in multiple 2D images captured along 100 µm segments, thereby generating 3-dimensional (3D) datasets. Parameters are assessed 3 months after injury, as we have previously observed ultrastructural changes to axons and myelin at this time point^11,12^.

## Results

### Changes in axonal and myelin ultrastructure along the length of axons

**Figure 1:**
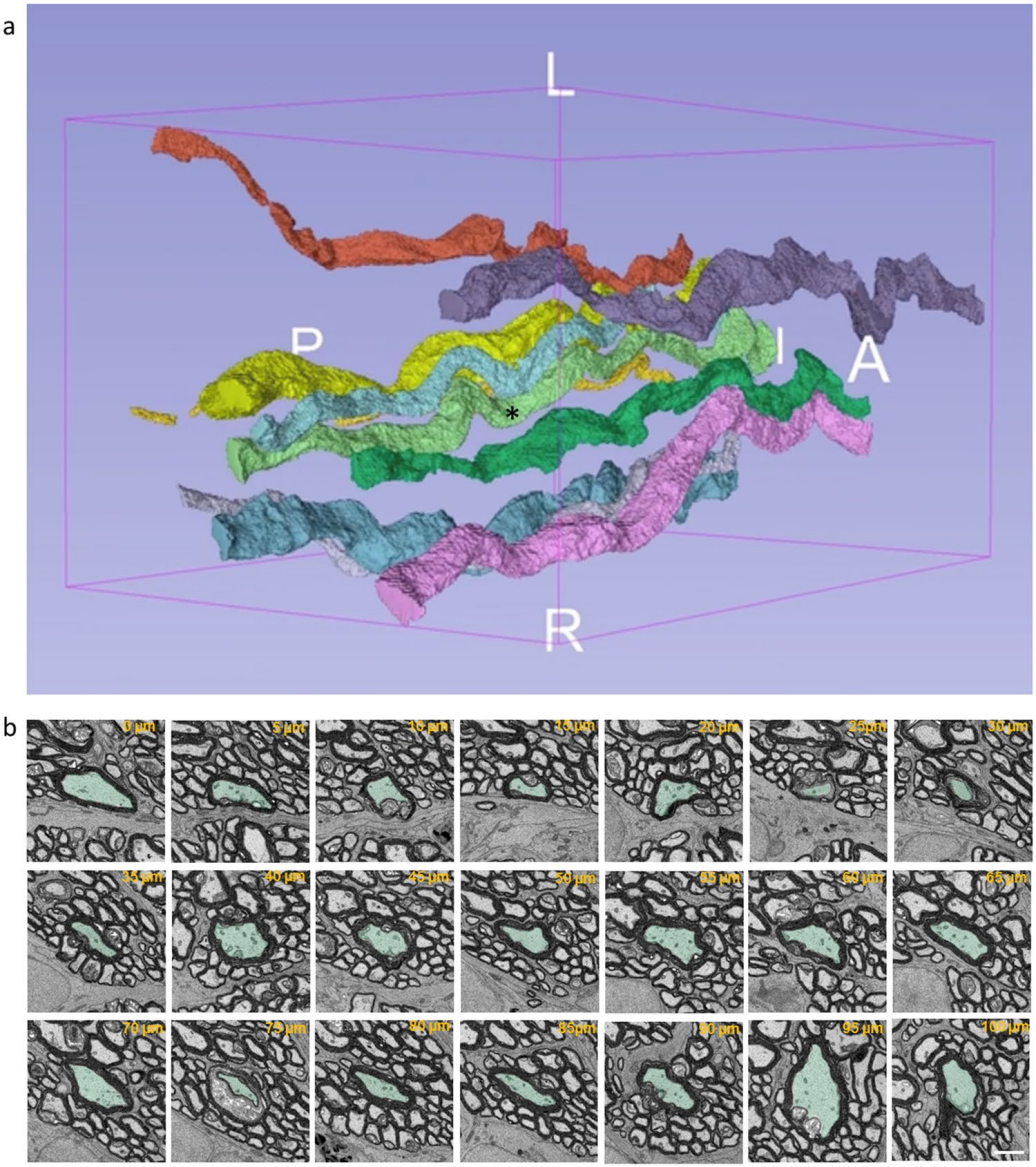
Representative axonal segmentation and 3D morphological representation of axons. (**a**) Representative 3D rendering of 10 randomly selected axons in optic nerve from a single normal animal. (**b**) Illustrates the variability in axonal morphology in a single axon indicated by * in (**a**), false-coloured in light green, at 5 µm intervals along its length. Scale bar = 2 µm.

**Figure 2:**
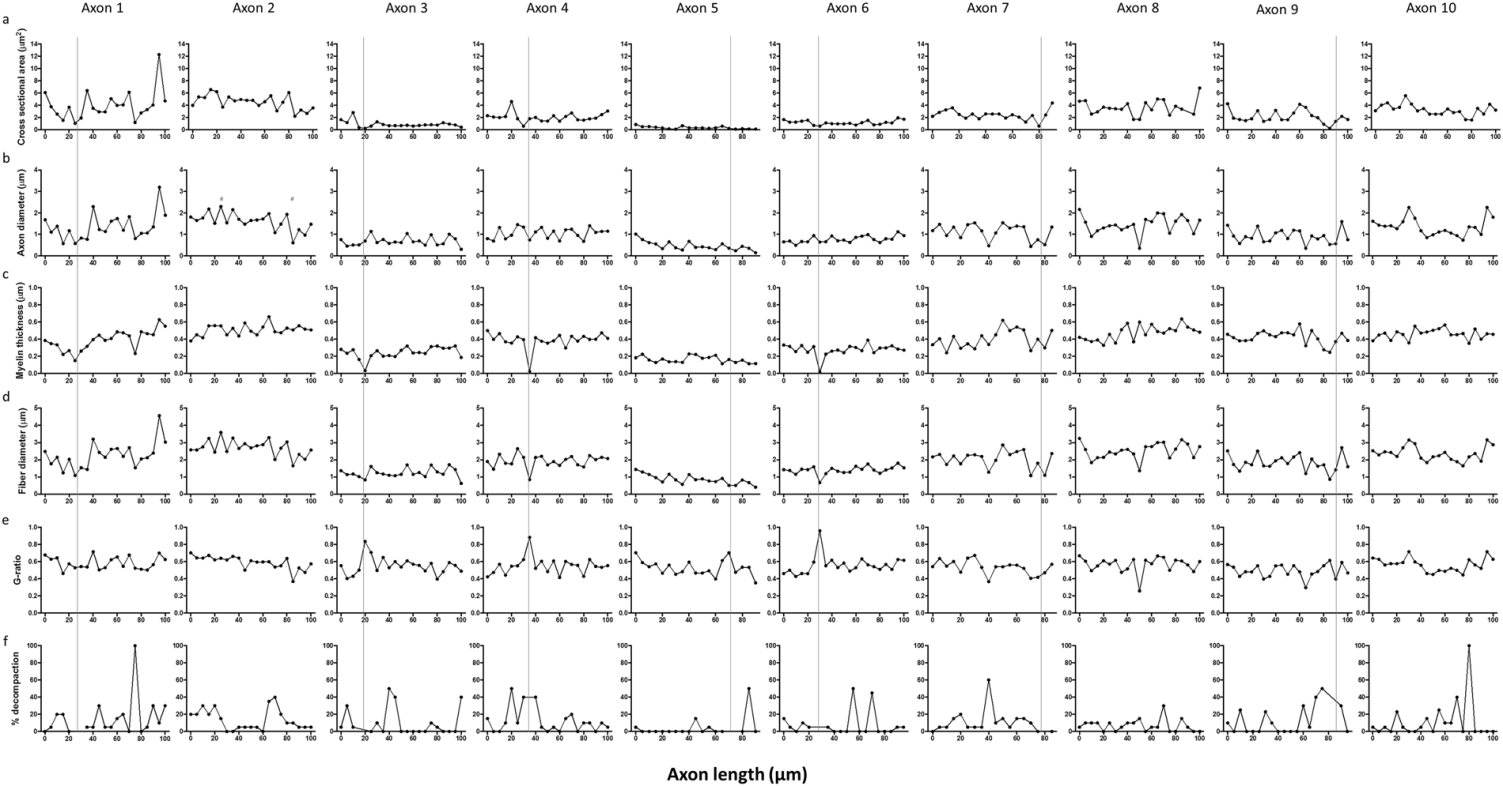
Morphological measurements made along the length of 10 randomly selected axons from optic nerve of a normal animal. Each column of graphs represents an individual reconstructed axon, showing measurements of (**a**) cross-sectional area, (**b**) axon diameter, (**c**) myelin thickness, (**d**) fiber diameter (**e**), G ratio, and (**f**) percentage myelin decompaction made from 2D images at 5 µm intervals along the length of that axon. Changes in the recorded outcomes are apparent along the length of individual axons. The grey lines represent the position of a node within the axon; in some axon segments the node is situated between data values. The red # represents the minimum and maximum axon diameter in segmented axon 1.

**Figure 3:**
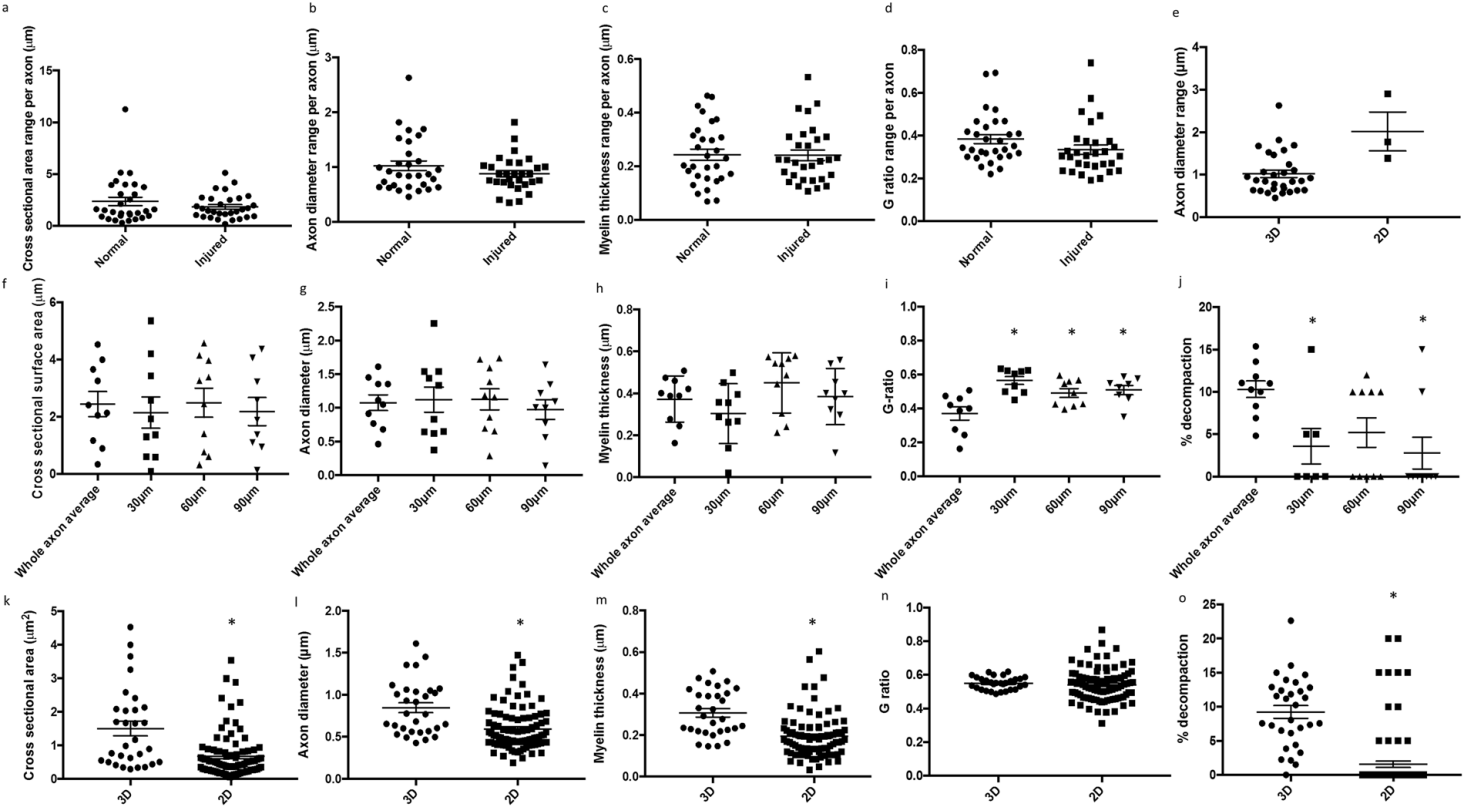
Range of values in 3D and comparisons between averages of values from individual reconstructed axons and 2D single sections for axonal and myelin morphology. The ranges of values for (**a**) cross sectional area, (**b**) axon diameter, (**c**) myelin thickness and (**d**) G-ratio obtained from 2D images along each individual reconstructed axon for normal and injured animals (n = 30 axons in each group). Each data point represents the range of an individual axon, derived from subtracting the minimum recorded value from the maximum for the given outcome. (**e**) Range in axon diameters derived from the 30 normal reconstructed individual axons compared to the range in a single 2D image from the same 3 normal animals. Averages derived from the 10 individual reconstructed axons analysed in 3D from a single randomly selected normal animal were compared to that animal’s averages derived from three single sections in 2D at 30 µm, 60 µm and 90 µm along the axons, for (**f**) cross sectional area, (**g**) axon diameter, (**h**) myelin thickness, (**i**) G-ratio and (**j**) percentage myelin decompaction. Comparisons between averages derived from 30 individual 3D reconstructed axons from normal animals, and averages from 30 randomly selected axons from a single section in 2D taken from each of the same 3 normal animals, for (**k**) cross sectional area, (**l**) axon diameter, (**m**) myelin thickness, (**n**) G-ratio and (**o**) percentage myelin decompaction. Data are expressed as scatter plots with the line representing the mean ± SEM. Differences between groups are indicated by *(p ≤ 0.05).

Axonal segments from normal and injured rat optic nerve were reconstructed at nanometer resolution using SBF SEM (Fig. 1a). It can be seen that an individual axon and the myelin surrounding it varies along the sampled 100 µm length (referred to hereafter as reconstructed in 3D), with 2D images of a single representative axon at 5 µm intervals revealing varying axonal diameter and myelin thickness (Fig. 1b). It is important to note, that in common with other 2D ultrastructural assessments of axons and myelin in the literature, axonal and myelin measures at any particular measurement point will be influenced by changes of axon direction relative to the sectioning plane, and by the angle of the tissue block. Indeed our data of Fig. 1 show that the axons move substantially and are often not perpendicular to the sectioning plane. Nevertheless, Supplementary Video 1 shows that the light green axon featured in Fig. 1b (asterisked) clearly has segments of smaller and larger axon diameter along the analysed length; other axons are similarly variable, showing that the variation in axonal diameter along individual axons is not solely an artefact of axon direction relative to the sectioning plane. Line graphs for cross sectional area, axonal diameter, myelin thickness, fiber diameter, G-ratio and percentage myelin decompaction measured at 5 µm intervals along axons were generated from 2D images at each measurement point and plotted for all reconstructed individual axons (n = 30 normal and n = 30 injured, see Supplementary Fig. S1). Representative graphs for 10 axons from a single uninjured animal are shown for cross sectional area (Fig. 2a), axonal diameter (Fig. 2b), myelin thickness (Fig. 2c), fiber diameter (Fig. 2d), G-ratio (Fig. 2e) and percentage myelin decompaction (Fig. 2f). While acknowledging the limitations of the varying orientation of the axon relative to the sectioning plane, virtually no outcomes remained constant along the length of an individual axon. For example, the maximum cross-sectional area of axon 1 in normal animal 1 was 12.30 µm^2^ and the minimum cross-sectional area was 1.04 µm^2^, observed 70 µm further along its length, giving a range of 11.26 µm^2^ (Fig. 2a). A similar observation was made for axonal diameter along a single axon, with Axon 2 in normal animal 1 having a diameter of 0.61 µm at its thinnest, in the section at 85 µm along its length, and a diameter of 2.3 µm at 25 µm along its recorded length (Fig. 2b, points indicated by red #). The pattern of substantial intra-axonal variability was observed in all assessed outcomes of ultrastructural morphology both in the normal and injured reconstructed axons, illustrated by the range of values (i.e. minimum subtracted from maximum) for each outcome measure (Fig. 3a–d).

Traditionally, ultrastructural outcomes have been quantified by assessing multiple axonal profiles in single 2D transmission electron microscopy images with a z-axis depth of approximately 50–100 nm. While numbers of axons quantified are low for such an analysis, we compared the range of values for axon diameter obtained from the 30 axons reconstructed in 3D from normal animals, to the range of values obtained from a single image from each of these three animals (Fig. 3e). The data sets overlap and were not statistically different from each other (t-test, F = 1.61, p = 0.16). Data from a single normal animal were further analysed, comparing the average value along each reconstructed axon, to values at defined 2D locations 30 µm, 60 µm and 90 µm along these same 10 axons. There were no observable differences between individual reconstructed axon averages and values at defined 2D locations for mean cross sectional area (Fig. 3f), axon diameter (Fig. 3g), or myelin thickness (Fig. 3h) (ANOVA, p > 0.05). However, comparisons of G ratio (Fig. 3i) (ANOVA, F = 7.85, p = 0.00) and decompaction (Fig. 3j (ANOVA, F = 4.32, p = 0.01) showed significant differences between the individual reconstructed axon averages and the mean values derived from 3 selected 2D sections for this animal.

A similar analysis was conducted comparing the average values from each of the 30 individual axons reconstructed in 3D from normal animals, to the traditional approach of assessing 30 randomly selected axons within a single section in 2D for each of these normal animals. There were statistically significant differences between individual axon averages and outcomes from single 2D sections for cross sectional area (Fig. 3k) (t-tests) (F = 3.15, p = 0.00), axon diameter (Fig. 3l) (F = 1.56, p = 0.00), myelin thickness (Fig. 3m) (F = 1.14, p = 0.00) and decompaction (Fig. 3o) (F = 1.32, p = 0.00). There were no differences in G-ratio when comparing 3D and 2D analysis outcomes (Fig. 3n) (F = 6.67, p = 0.43).

### Changes in axon and myelin ultrastructure with injury

**Figure 4:**
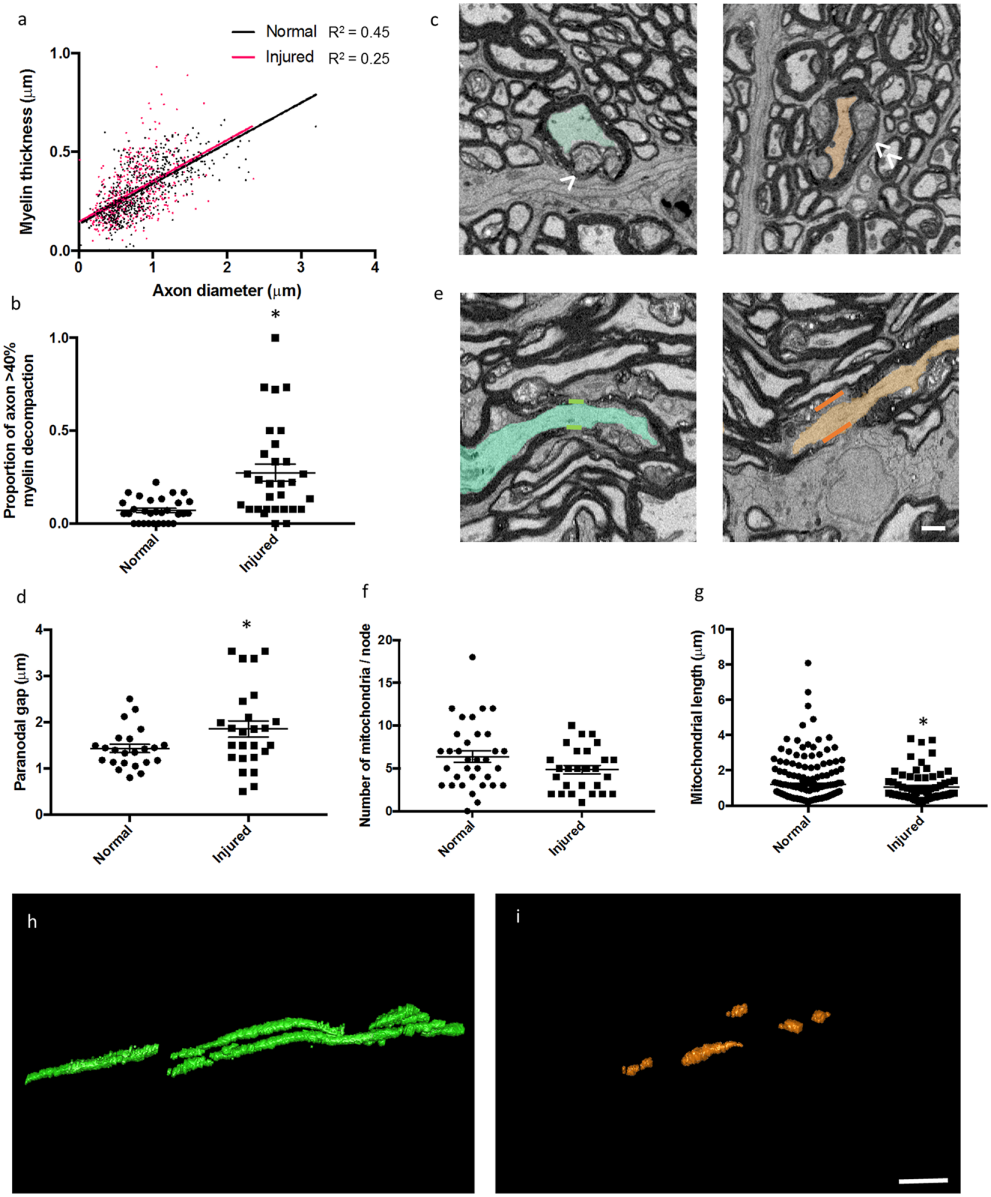
Quantification of myelin and mitochondrial changes in reconstructed axons in 3D following injury. (**a**) Correlation between axon diameter and myelin thickness generated from values in 2D from sections 5 µm apart along the axons; normal optic nerves R^2^ = 0.449, whilst after injury R^2^ = 0.249. Linear regression line for normal fits the equation: y = 0.20 × +0.13, and injured: y = 0.21 × +0.15; n = 30 axons in each group. (b) Myelin decompaction was quantified for normal and injured axons at 5 µm intervals along the length of each reconstructed axon. Individual reconstructed axons classified as having >40% myelin decompaction in single sections at 5 µm intervals were given a ‘point’ and total points of decompaction along the axon were expressed as a proportion of the measured length of the axon. (**c**) Representative image of a myelin segment <40% decompaction (green, arrow) and >40% decompaction (orange, double arrow) from transverse sections. Myelin was considered decompacted by the presence of light grey regions located between the concentric darker, compact myelin lamellae. (**d**) Paranodal gap was increased following injury to the optic nerve. (**e**) Representative image of paranode before (green) and after (orange) injury; there is an elongation of the paranodal gap, shown visualizing the 3D dataset longitudinally. The (**f**) number and (**g**) length of mitochondria within all nodes of Ranvier in the 30 axons examined per experimental group were quantified for normal and injured axons. Figures are expressed as scatter plots with the line representing the mean ± SEM. Differences between experimental groups are shown by *(p ≤ 0.05). Representative images of mitochondria from normal (**h**) and injured optic nerve (**i**) are shown. Scale bar for c and e = 1.5 µm; for h and i = 1 µm.

The relationship between axonal diameter and myelin thickness is thought to reflect signal conduction velocity along an axon^16^. Assessment of the correlation between axonal diameter and myelin thickness at measurement points along 3D reconstructed axons revealed differences as a consequence of injury (Fig. 4a). In normal animals, the correlation between axon diameter and myelin thickness had an R^2^ = 0.45. This was reduced after injury to R^2^ = 0.25, indicating a change in the relationship between axonal diameter and myelin thickness (Fig. 4a). Percentage myelin decompaction was assessed in sections at 5 µm intervals along each individual reconstructed axon and assigned a ‘point’ for every section at which greater than 40% of the axonal circumference showed decompaction of the associated myelin (Fig. 4b,c). 40% was chosen as the threshold for myelin decompaction, as this is the value that provides the greatest differential between normal and injured optic nerves. To correct for the differences in lengths of axons measured, percentage decompaction values were expressed per measured axon length. Injured optic nerves showed a significantly higher proportion of single sections with >40% decompacted myelin sheath compared to normal animals (t-test, F = 15.95, p = 0.00) (Fig. 4b). Additionally, assessment of paranodal gap length revealed significant increases in the injured optic nerve axons compared to normal (t-test, F = 4.10, p = 0.04) (Fig. 4d,e). Not all of the sampled axons have Nodes of Ranvier within the 100 µm of analysis, and therefore the number of data points are correspondingly reduced.

### Changes in mitochondria with injury

The number and length of axonal mitochondria located at the node of Ranvier (within 10 µm of the node) were also examined. Compared to normal, there was no difference in the number of mitochondria located at the node of Ranvier following injury (t-test, F = 2.23, p = 0.07) (Fig. 4f). However, the mitochondrial length was significantly shorter in injured optic nerve compared to normal (t-test, F = 2.54, p = 0.01) (Fig. 4g). Note that some nodes have no mitochondria and others have multiple mitochondria, hence the number of data points is not necessarily 30.

### Average changes along individual axons

**Figure 5:**
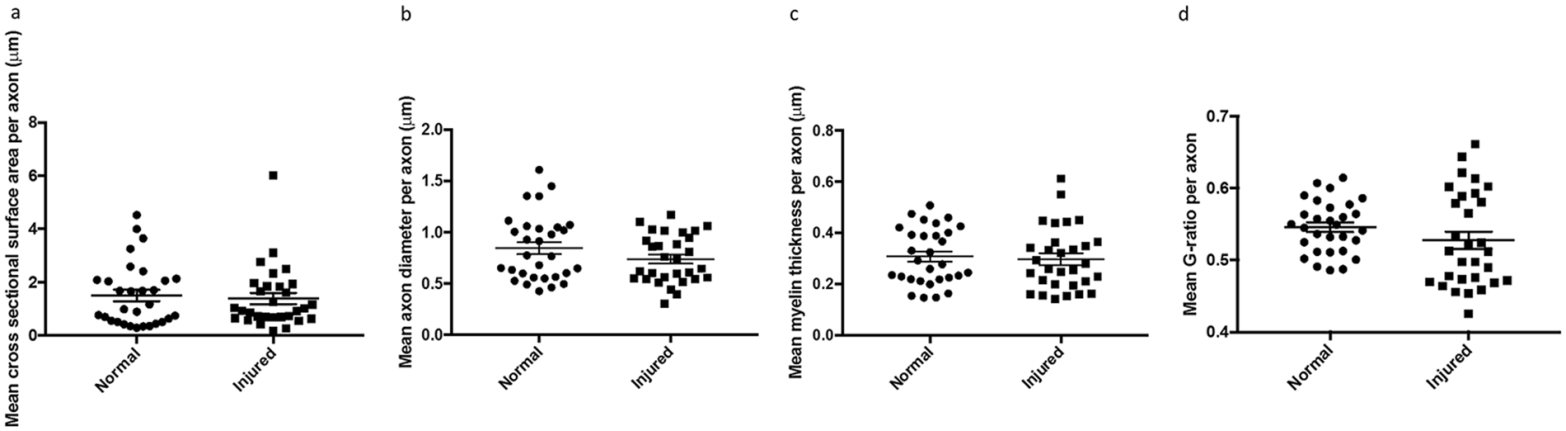
Mean measurements from individual reconstructed axons for (**a**) cross-sectional surface area, (**b**) axonal diameter, (**c**) myelin thickness and (**d**) G ratio in normal and injured animals; n = 30 axons in each group. Each data point represents the mean for an individual axon derived from averaging 2D measurements made at 5 µm intervals. Data are expressed as scatter plots with the line representing the mean ± SEM. Differences between groups are indicated by *(p ≤ 0.05), none observed.

Cross sectional area, axonal diameter, myelin thickness, fiber diameter, G ratio and percentage decompaction were recorded at 5 µm intervals for each individual reconstructed axon analysed. Measurements recorded over the length of each axon were averaged, giving a single mean value per axon, and these mean values were used to compare averages in normal and injured animals (n = 30/experimental group). Using this type of analysis, there were no differences with injury in mean cross sectional area (t-test, F = 1.03, p = 0.70) (Fig. 5a), axonal diameter (t-test, F = 1.86, p = 0.15) (Fig. 5b), myelin thickness (t-test, F = 1.24, p = 0.72) (Fig. 5c) or G-ratio (t-test, F = 1.03, p = 0.13) (Fig. 5d). The variability introduced by differing axon direction relative to sectioning plane, together with the relatively small number of axons analysed for this comparison, likely contributed to the lack of differences observed with injury.

## Discussion

### Changes in axonal diameter in normal nerve

Using SBF SEM, the current study revealed changes in axon caliber and myelin sheath thickness along the length of individual axons in the optic nerve of normal animals and following injury. Although variation along the lengths of axons has been previously investigated in unmyelinated tracts of the peripheral nervous system and retina, the spatial resolution was insufficient to enable investigation of intricate changes to axon diameter along individual axons^21,22^. Variability in axonal diameter is also observed in the auditory system, but between daughter branches from a parent axon, not within single axon branches^23,24^. Additionally, there was no quantification of changes to the myelin sheath over the length of the axon in these studies. Using high resolution SBF SEM, it was possible to discern differences in ultrastructure along axons and their associated myelin sheath. The 500% increase in fiber diameter of axon 1 (Fig. 2d) exemplifies the degree of change possible along an individual axon, while bearing in mind the possible variability due to changing axon orientation relative to the sectioning plane.

Changes in morphology along the length of axons has been reported in studies assessing the visual system^21,25^, auditory system^24,26^ and peripheral nervous system^27–29^, particularly at the nodes of Ranvier, where axonal diameter is known to decrease^30^. Light microscopic assessment of intra-retinal axon diameter indicated differences in axonal diameter along the length of the axon^21^; changes to myelin sheaths were not considered^21^. In the mature auditory system of the gerbil, there are differences in the internode length and axonal diameter when comparing the ipsilateral and contralateral branches of the same parent anterior ventral cochlear neuron, exemplifying morphological changes in axon diameter within an individual neuron^26^. Scanning block face serial EM has revealed morphological and mitochondrial dynamics to axonal evulsions and associated astrocytes in normal optic nerve head^31^ and following optic nerve crush injury^32^ in mouse. In the current study, we observe intra-axonal changes in the diameter of axons of the optic nerve, which may potentially reflect previously observed variability in conduction velocity between the optic tract and the optic nerve^25^. RGCs are classified based on their morphological and electrophysiological properties with different sized RGC somata thought to be associated with different sized axons^33–40^. Generally, there is a trimodal distribution of RGCs, which in the rat have been classified as large (L-), medium (M-) and small (S-) cells^40^. RGCs have been classified into subtypes using electrophysiology^40^ and retrograde labelling of somata and associated dendritic fields^41,42^, with a few studies providing axon diameter as a defining characteristic of these cells^43,44^. The New World primate (*Callithrix jacchus*) demonstrates variability in axonal diameter along their intra-retinal length, with the rate of change related to RGC class and the initial size of the axon^21^. Interestingly, the range of axon diameters measured from a single axon in the current study spanned multiple axonal diameter criteria for the trimodal RGC classification of other studies, including in rat^21,43–46^ (Table 1). It is possible that studies to date assessing variation in axonal diameter and myelin structure using single sections may be confounded by variability along individual axons^21,43–46^. The variability in axon diameters observed in the 2D component of the current study and reported in the literature may arise from both variation along individual axons and varying axon diameters in functionally distinct RGC types, both potentially working to reduce spatiotemporal dispersion along the visual pathway^47^.

**Table 1:** Comparison of range of values for axon diameter along an individual axon in the current 3D SEM study, compared to axon diameter outcomes reported in the literature for a range of animals including rat. PVG is Piebald Virol Glaxo.

Paper	Animal	Axon diameter
Current study	PVG rat	Example individual axon range: Small axon: 0.49–1.2 µm Large axon: 0.57–3.19 µm
Reese^46^	Hooded rat	Fine: ≤2 µm Coarse: >2 µm
Guy *et al*.^44^	Strain-13 guinea pig	Small = ≤ 2 µm Medium = 0.8–2.0 µm
Fitzgibbon and Funke^45^	Cat	Temporal retina modal peaks: 0.6–0.8 µm 1.4–2.1 µm 3.3 µm
Drenhaus, von Guten and Rager^43^	Tupaia cat	Mean diameters: Small = 0.55 µm Medium = 0.88 µm Large = 1.30 µm
Walsh, Fitzgibbon and Ghosh^21^	Common marmoset (*Callithrix bacchus*)	Axon diameter range of RGCs: Parasol = 0.8–1.8 µm Midget = 0.7–1.3 µm Hedge = 0.6–1.0 µm SBS – 0.7–1.1 µm

### Changes in myelin sheath thickness in normal nerve

Variations in myelin thickness may also provide a mechanism for temporal adjustment or complex transformations of neural information^24^. In the peripheral nervous system, differences in myelin sheath thickness were found along ventral motor neuron axons^27,28^. The variability in the thickness of the myelin sheath within individual internodes could be accounted for by changes in the number of intraperiodic lines. In the developing CNS, it has been found that the number of oligodendrocyte wraps is greatest at the site where the oligodendrocyte process is connected to the growing myelin sheath, and this gradually decreases towards the extremities of the internode^48,49^. Reductions in myelin thickness at the extremities of the internode were not observed in the current study (Fig. 2), suggesting that internodes in the adult CNS fully ensheath axons along their whole length.

Comparisons of data derived from 2D analysis of 90 axons to 3D analysis along 30 individual axons from normal animals revealed differences in outcomes for cross sectional area, axon diameter, myelin thickness and decompaction. While the numbers of axons assessed for this comparative analysis are relatively low, it is important to consider that outcomes derived from 2D analyses of axonal and myelin morphology may not reflect outcomes if a similar number of axons were analysed in 3D. Further studies comparing outcomes for a larger number of axons would be required to definitively address this point.

### Myelin decompaction with injury

Damage to, or loss of, the insulating myelin sheath is a contributor to functional impairment associated with neurotrauma and demyelinating disease^50,51^. Increased decompaction as a consequence of injury reflects our previous findings in the optic nerve, manifesting as myelin sheath decompaction and disruption to the node of Ranvier and paranodes^11,12^. It has been suggested that myelin decompaction is an artefact of the fixation process for electron microscopy^52^. However, the presence of distinct, loosely wrapped myelin in proteolipid protein (PLP)-deficient mice suggests decompaction occurs following changes to PLP responsible for the compaction of adjacent lamellae^53^. It has been demonstrated that there is a high concentration of reactive oxygen species including hydroxyl radicals^54^ and nitric oxide^55^ following traumatic injury to white matter. These species may disrupt proteolipid proteins *via* lipid peroxidation, as well as the myelin proteins located between myelin lamellae, leading to a loosening of lamellae and an increase in myelin thickness, with associated functional deficits^56–58^. The average space between adjacent myelin lamellae is approximately 3 nm^59^ and therefore individual lamellae were unable to be resolved using SBF-SEM, which has a feature resolution of 11.5 nm. Nevertheless, decompaction of the myelin sheath was observed in both the normal optic nerve and to a much greater extent, the injured optic nerve. Previous quantification of myelin decompaction using single sections following partial transection of the optic nerve showed 12–15% of axon populations with large scale myelin decompaction (15% or greater decompaction of the myelin sheath surrounding the axon)^11^. By comparison, quantification using 3D EM in the current study reveals a much higher proportion of decompacted myelin segments following injury, highlighting the importance of selecting analytical techniques that can effectively quantify the outcome measure of interest. Serial block face scanning EM has been used to good effect in studies of frog metamorphosis revealing shortening of myelin segments^60^ in the optic nerve, and demonstrating the power of the technique.

Alterations to the length of the paranodal gap may also be associated with functional impairment^12^. Recent findings show that alterations in node of Ranvier length is sufficient to produce large variations in action potential conduction speed, equivalent to adding or removing a single lamella of myelin^17^. The changes to nodal ultrastructure after injury could potentially impede the timing of signals to the relevant areas of the brain. The observed changes to myelin and nodal structure are likely key contributors to the related impairments in function seen in this model, where visually guided behaviour is reduced at 3 months following partial optic nerve transection^11^.

### The relationship between axon diameter and myelin sheath thickness

Analysis of 3D reconstructed axons indicated a reduced correlation between axon diameter and myelin thickness with injury. It is generally accepted that there is a positive relationship between axon caliber and associated myelin sheath thickness^61^. The changes in G-ratio along the length of individual axons, both normal and injured, imply a heterogeneous conductance of the action potential along the axonal length. Variability in signal propagation velocity has previously been suggested for action potentials travelling between the optic nerve and optic tract^25^. It was assumed that the variation in conduction velocity takes place in different parts of the same axons, but this was not experimentally determined. The morphological changes along the length of axons that we show here imply there may be subtle changes to axon diameter and myelin thickness which are capable of regulating conduction velocity. Axons are generally assumed to be cylinders of invariant diameter in quantification studies, and conduction velocity of nerve fibers has been estimated using axonal diameters obtained from single 2 dimensional sections^10,11^. The reconstructions of myelinated axons in the current study reveal that virtually all axons have a highly irregular shape. The irregular morphology of axons and their associated myelin sheaths raise questions about the validity of 2D analyses and also suggests that electrical properties of axons may be more heterogeneous than previously expected.

The earliest change in spared axons following traumatic insult is swelling of axonal mitochondria^62^. High intracellular concentrations of Ca^2+^ result in sequestering of these ions into mitochondria, leading to mitochondrial failure and reduced ATP production^63^, generation of free radicals and apoptotic cell death^64^. Neurons have a high metabolic demand for the maintenance of ionic gradients across cell membranes and for neurotransmission^64^, and depend on mitochondrial function and oxygen supply to meet these energy needs^65,66^. Within axons, mitochondria typically accumulate around areas of highest energy demand such as the nodes of Ranvier, where the production of ATP is necessary to maintain activity of the energetically demanding Na^+^/K^+^-ATPase ion pumps^67,68^. Mitochondria change their morphology according to the energy status of the cell in processes referred to as fusion and fission^69^. Following spinal cord injury, there is an increased prevalence of fragmented mitochondria, due to alterations in fission and fusion genes^70^; as such, altered 3D mitochondrial morphology is reflective of axonal pathology. Mitochondria are an integral component of the response to oxidative stress as a consequence of increased reactive species, responding with increased fission^71^. In the current study, while the numbers of mitochondria remained similar to normal tissue following injury, their length decreased, indicating increased fission accompanied by autophagy, as previously reported^72^, thereby keeping numbers similar to those in normal tissue.

## Conclusions

The development of SBF SEM offers an unparalleled tool for assessment of axon and myelin ultrastructure, as well as the distribution of intra-axonal organelles. The time-consuming, costly nature of 3D SEM analysis requires experimental planning to ensure the most appropriate analysis is performed for the outcome of interest. Future studies will benefit from incorporating both high throughput single section 2D analysis and 3D assessment of tissue samples to attain an understanding of the intricate changes occurring within tissue following injury and in other disease conditions. The capacity to assess structure using 3D datasets presents the opportunity for a more comprehensive understanding of the structural changes resulting from CNS pathophysiology.

## Methods

### Animals

All animal procedures were carried out in accordance with the guidelines approved by The University of Western Australia Animal Ethics Committee, approval number RA3/100/673, and adhered to the National Health and Medical Research Council Australian Code of Practice for the care and use of animals for scientific purposes. Adult female piebald-Virol-Glaxo (PVG) rats were obtained from the Animal Resources Center (Murdoch, Western Australia) and housed under temperature controlled conditions on a 12-hour light/dark cycle, with access to rat chow and water *ad libitum*. Animals were randomly assigned into one of two groups; uninjured 3 months (n = 3) and injured 3 months (n = 3). The numbers of animals per group were appropriate given the nature of the highly intensive ultrastructural characterizations along the length of optic nerve segments, and are in line with other published electron microscopy based studies based on two dimensional analyses^1,73^.

### Surgical procedures

Partial transection of the optic nerve was conducted as previously described^12^. In brief: animals were anaesthetised with xylazine (10 mg/kg IP ilium xylazil; Troy Laboratories) in combination with ketamine (50 mg/kg Ketamil, Troy Laboratories). A midline incision was made along the skin overlying the skull, the skin was retracted, access to the right optic nerve was achieved by deflecting the Harderian lachrymal gland immediately behind the eye. The nerve parenchyma was exposed by making an incision in the dura mater using ophthalmic scissors. The dorsal aspect of the right optic nerve was incised to a depth of 200 µm, 1 mm behind the right eye using a diamond radial keratotomy knife. The depth of the cut was limited by the protrusion of the blade past the surrounding guard. Post-operative analgesia was provided once (subcutaneous injection of 2.8 mg/kg Carprofen (Norbrook Australia, Pty. Ltd; Victoria, Australia)). Three months later, animals were euthanized using Lethabarb (sodium pentobarbitone 850 mg/kg plus sodium phenytoin 125 mg/kg; Provet, Western Australia, Australia). Completely normal animals were similarly housed for the three month period and were used as controls; the decision was made not to use sham injured animals as controls as we were seeking to identify changes in completely normal animals, where there was no chance of sham injury changing outcomes.

### Preparation of optic nerves

Following euthanasia, animals were transcardially perfused with 0.9% saline followed by phosphate buffered 2.5% glutaraldehyde (Sigma-Aldrich; St Louis, Missouri, United States)/2% paraformaldehyde (Sigma-Aldrich; St Louis, Missouri, United States), pH 7.2. Optic nerves were dissected and post-fixed in 2.5% glutaraldehyde/2% paraformaldehyde in phosphate buffer for an additional 2 hours. Optic nerves were cut into 2 mm lengths using a razor blade and stored in Sorenson’s buffer (pH 7.4) at 4 °C, this length encapsulated the whole injury site in the injured optic nerves. Samples were prepared for serial block face SEM (SBF SEM) using a modification of the protocol previously described by Deerinck *et al*.^74^. Optic nerves were washed in cold 0.1 M phosphate buffer pH 7.4 (3 × 5 minutes), followed by post-fixation in a reduced osmium solution containing 2% osmium tetroxide (ProSciTech, QLD, Australia), 1.5% potassium ferrocyanide (Univar, IL, USA) in 0.1 M phosphate buffer pH 7.4 for 1 hour at room temperature. Nerves were subsequently washed with milliQ water (3 × 5 min) before incubating with freshly prepared and filtered 1% aqueous thiocarbohydrazide (ProSciTech, QLD, Australia) for 20 min at room temperature. Nerves were then treated with 2% osmium tetroxide for 30 minutes at room temperature after washing with milliQ water (3 × 5 minutes), followed sequentially by incubation in 1% aqueous uranyl acetate overnight protected from light at 4 °C. The samples were then incubated in Walton’s lead aspartate solution (0.66%) for 30 minutes at 60 °C followed by a dehydration series, consisting of 30%, 50%, and 70% ethanol 2 × 5 minutes, 90%, 100% ethanol 2 × 10 minutes, and a 10-minute incubation in 100% acetone. Optic nerves were then placed in the following concentrations of hard grade Procure 812 resin (ProSciTech, QLD, Australia): 25% for 3 hours, 50% overnight, 75% for 4 hours, 100% overnight (twice) and embedded in fresh 100% Procure 812 resin and allowed to cure for at least 48 hours at 60 °C^74^.

Resin embedded optic nerves were trimmed down to approximately 1 mm^3^ and mounted on aluminum specimen pins (Gatan, Pleasanton, CA) using cyanoacrylate glue. Silver paint (Ted Pella) was used to electrically ground the exposed edges of the tissue block to the aluminum pin. The entire surface of the specimen was sputter coated with a thin layer of gold (10 nm).

### SBF-SEM imaging

Serial images of the resin embedded sample block face were generated by repeated cycles of sectioning with a diamond knife and imaging. Images were acquired using a variable pressure, field emission scanning electron microscope (Sigma VP, Carl Zeiss) equipped with a Gatan 3View 2XP. Backscattered electron images were collected at a fixed working distance of 4.4 mm using the following imaging parameters: 3 kV, 28 Pa, 30 μm aperture, 10000 × 10000 pixels, XY pixel size 5 nm, Z pixel size (slice thickness) 50 nm and pixel dwell time 1.5 μs. Sets of 2000 images at 50 nm steps were obtained at a 5 nm/pixel resolution, producing whole datasets of 50 × 50 µm in the X, Y-plane and 100 µm in the Z-plane. Imaging conditions were chosen to ensure maximum axon sample length whilst still discerning mitochondria. At 5 nm/pixel resolution, mitochondria were identified as dark, electron-dense oval-shaped organelles with a diameter over 0.1 µm.

### Image analysis

Axons, myelin and mitochondria were measured using Microscopy Image Browser software (University of Helsinki)^75^. 10 axons of varying diameter were randomly selected within the 50 × 50 µm field of view for segmentation and were traced from the first section in which they appeared, until they were no longer within the field of view. Measurements of axon diameter, myelin thickness, fiber diameter (axon and myelin diameter), G ratio (axon diameter/fiber diameter) and percentage of axon diameter with decompacted myelin were performed on 2D images at 5 µm intervals along the length of the axons using FIJI image analysis software. The minimum axon diameter and myelin thickness were used for measurements to minimize variability within the data set due to movement of axons relative to the sectioning plane. Myelin was considered decompacted by the presence of light grey regions located between the concentric darker, compact myelin lamellae. Percentage myelin decompaction of total axonal diameter was calculated by estimating the percentage proportion of the total axonal circumference occupied with decompacted myelin in 2D sections. Mitochondrial length was assessed by measuring the total length of individual mitochondria. The starting and end points were determined from when the mitochondria was first in view in the z-plane, until it was no longer visible.

### Data presentation and statistical analysis

Individual data points were displayed as scatter or box and whisker plots for all data values (myelin thickness vs axon dimeter scatter plot) or for each of the thirty axons within each experimental group, with median ± SEM displayed (10 axons/animal). Discrete data (e.g. number of mitochondria/node) were displayed as incidence for all axons within a group. Statistical analyses were conducted using SPSS statistical software (IBM). Normality of data was assumed and homogeneity of variances was tested using Levene’s test. Values from uninjured nerve were compared to 3 months injured nerve using unpaired t-tests. Comparisons between mean axon length averages and single slice results were analysed using repeated measures ANOVA with Tukey’s or Dunnett post-hoc tests as appropriate.

### Data Availability

All data are available in Supplementary Fig. S1 and in alternative formats upon request to the authors.

## Electronic supplementary material

Supplementary Material

Supplementary Video 1
