# Supplementary material for: Three dimensional electron microscopy reveals changing axonal and myelin morphology along normal and partially injured optic nerves
Source: Sci Rep. 2018 Mar 5;8:3979. doi: 10.1038/s41598-018-22361-2 (PMC5838102; doi:10.1038/s41598-018-22361-2)
Supplement: Supplementary file 1 — Supplementary Material [file 41598_2018_22361_MOESM1_ESM.pdf]

Supplementary Material

Three dimensional electron microscopy reveals changing axonal and myelin morphology along normal and partially injured optic nerves

Marcus K. Giacci[1], Carole A. Bartlett[1], Minh Huynh[3], Matt R. Kilburn[2], Sarah Dunlop[1] and Melinda Fitzgerald[1,4,5]

[1]*Experimental and Regenerative Neurosciences, School of Biological Sciences,*

[2]*Centre for Microscopy, Characterisation, and Analysis. The University of Western Australia, 35 Stirling Hwy, Perth 6009 Western Australia, Australia.*

[3]*Australian Centre for Microscopy and Microanalysis. The University of Sydney, City Road, Sydney 2006 New South Wales, Australia.*

[4]*Curtin Health Innovation Research Institute, Curtin University*

[5]*Perron Institute for Neurological and Translational Science, Sarich Neuroscience Research Institute, 8 Verdun St, Nedlands 6009 Western Australia, Australia*

Supplementary Figure S1: Morphological measurements made along the length of 10 randomly selected axons from normal optic nerve and regions of secondary degeneration following partial optic nerve transection. Axons were from normal (a, b) and injured (c, d, e) animals. Similar to Figure 2 in the main paper, each column of graphs represent individually reconstructed axons, showing measurements of cross-sectional surface area, axon diameter, myelin thickness, fiber diameter, G ratio and percentage myelin decompaction. Measurements were made at 5um intervals along the length of that axon. Changes to the recorded outcomes are apparent along the length of individual axons. Grey lines represent the position of the node within the axon where present; in some axon segments the node is situated between data values.

a

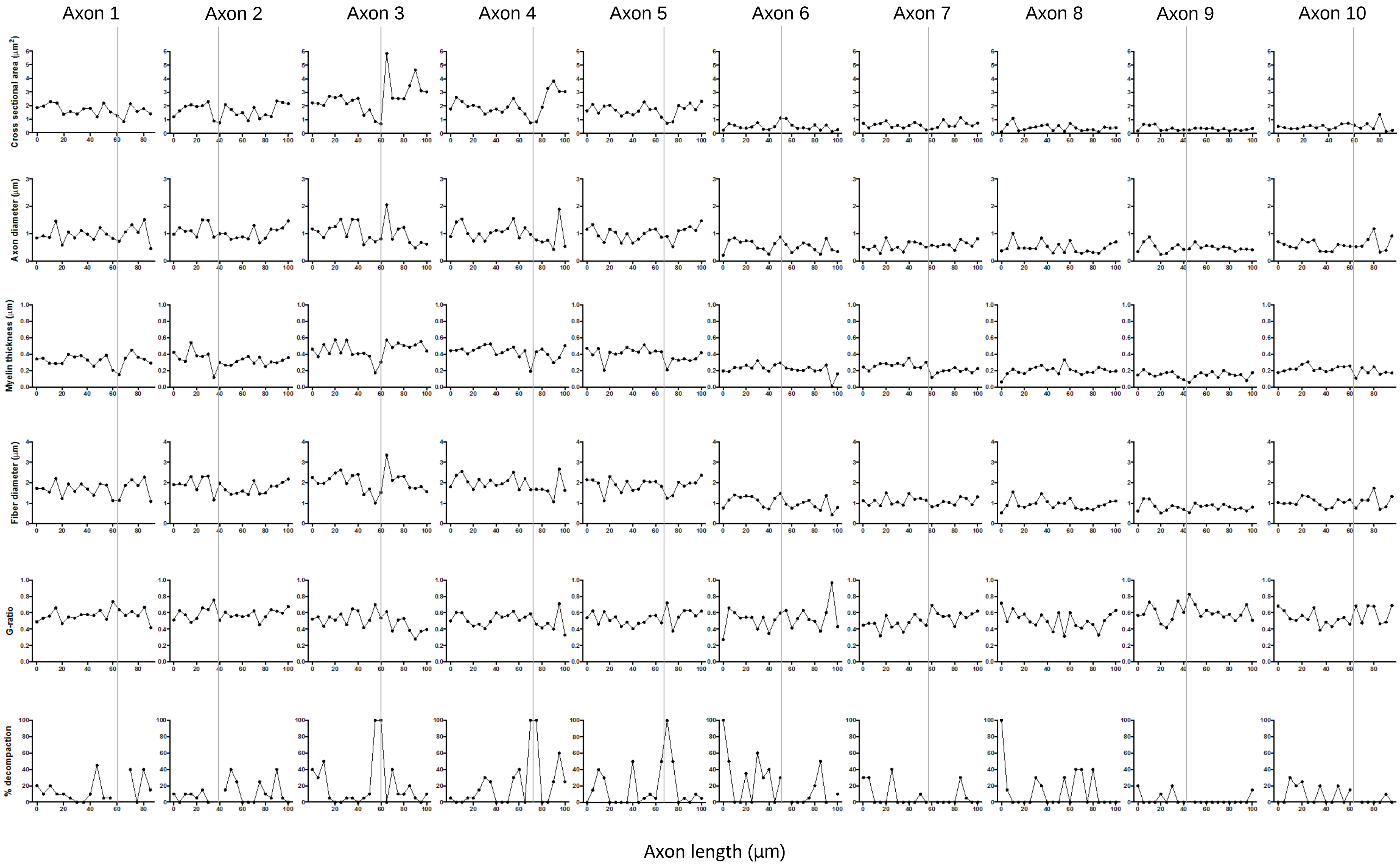
Axon 1
Axon 2
Axon 3
Axon 4
Axon 5
Axon 6
Axon 7
Axon 8
Axon 9
Axon 10
Cross sectional area (μm^2)
Axon diameter (μm)
Myelin thickness (μm)
Fiber diameter (μm)
G-ratio
% decompaction
Axon length (μm)

b

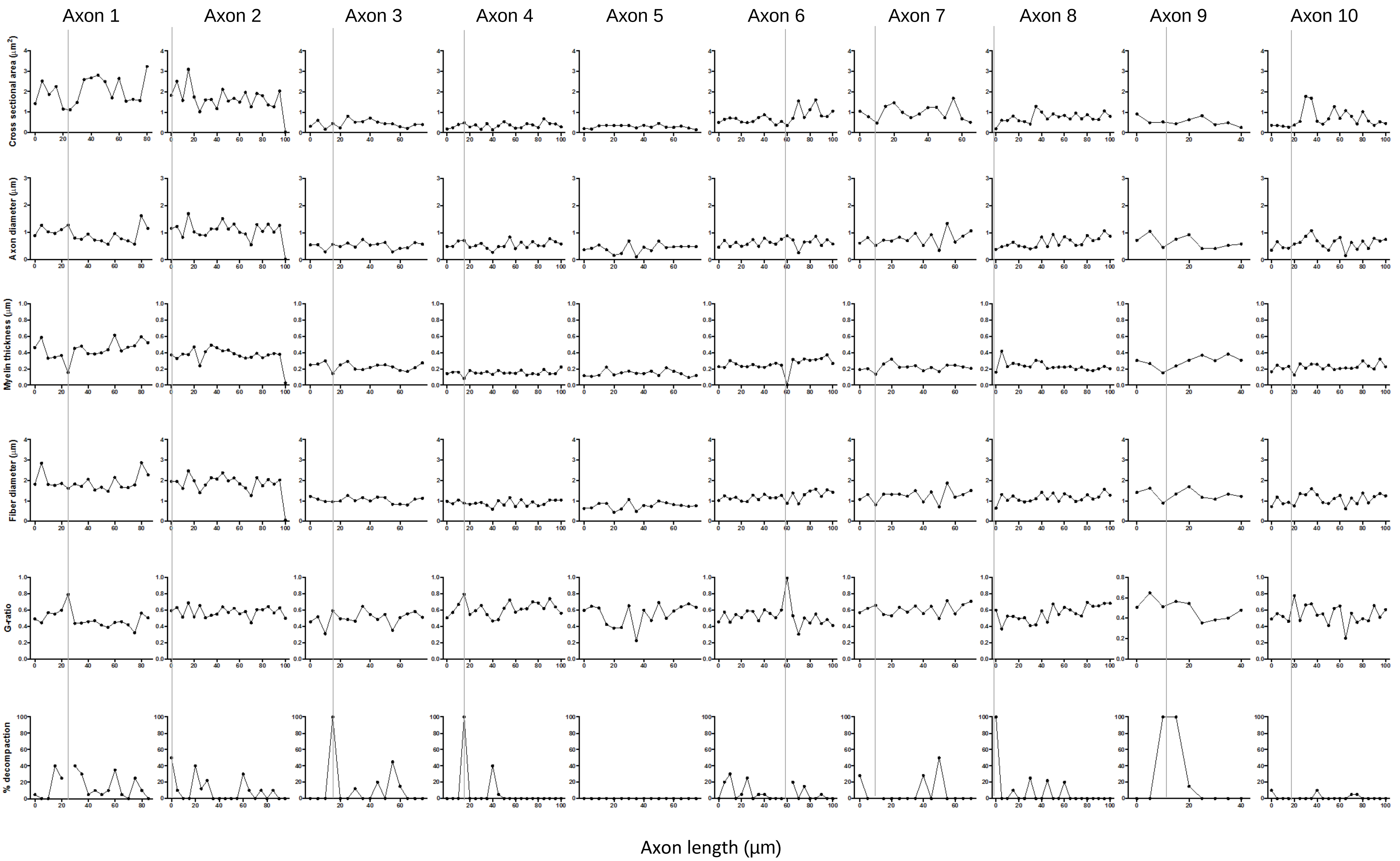
Axon 1
Axon 2
Axon 3
Axon 4
Axon 5
Axon 6
Axon 7
Axon 8
Axon 9
Axon 10
Cross sectional area (µm²)
Axon diameter (µm)
Myelin thickness (µm)
Fiber diameter (µm)
G-ratio
% decompaction
Axon length (µm)

c

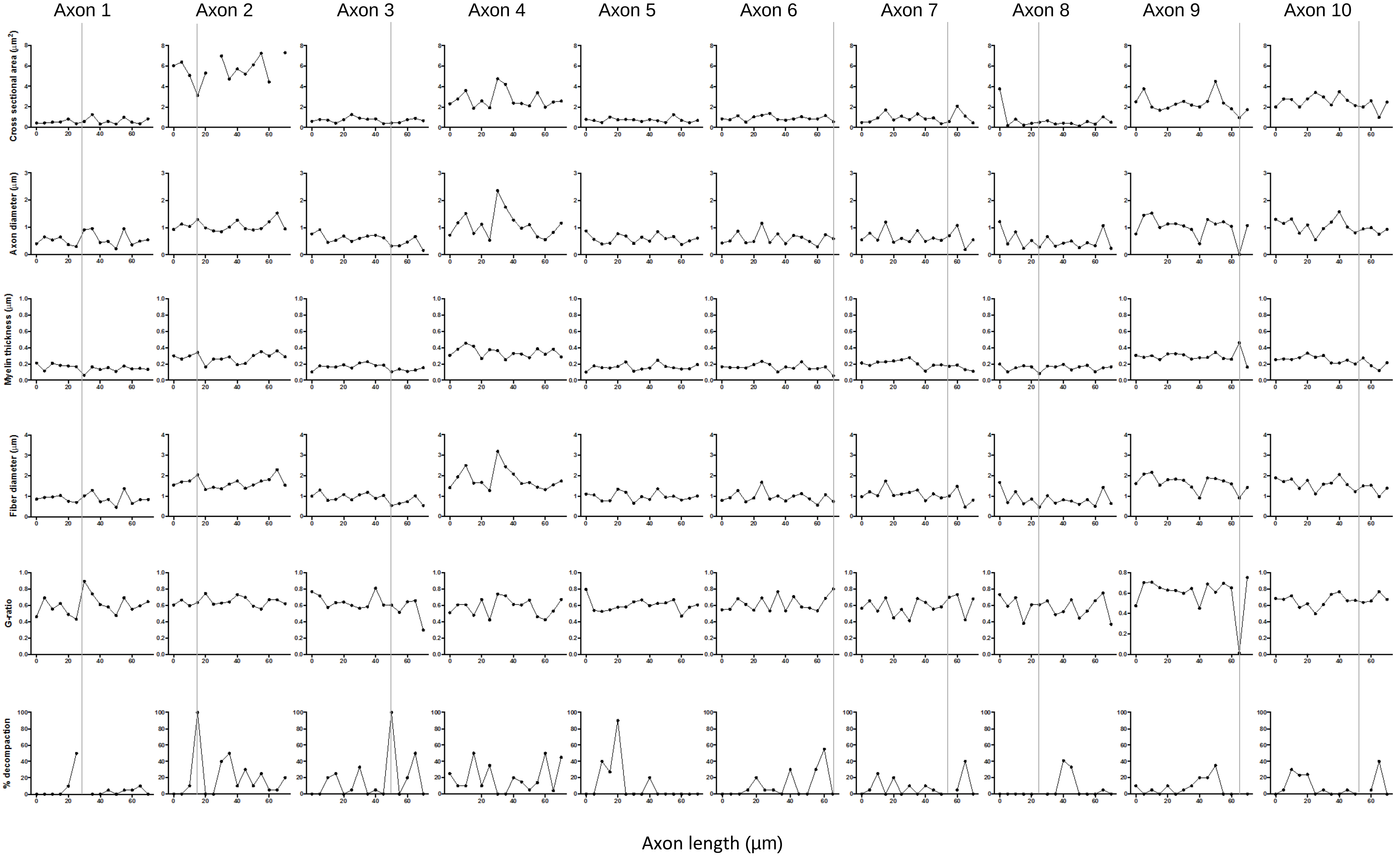

Axon 1
Axon 2
Axon 3
Axon 4
Axon 5
Axon 6
Axon 7
Axon 8
Axon 9
Axon 10
Cross sectional area (μm²)
Axon diameter (μm)
Myelin thickness (μm)
Fiber diameter (μm)
G-ratio
% decompaction
Axon length (μm)

d

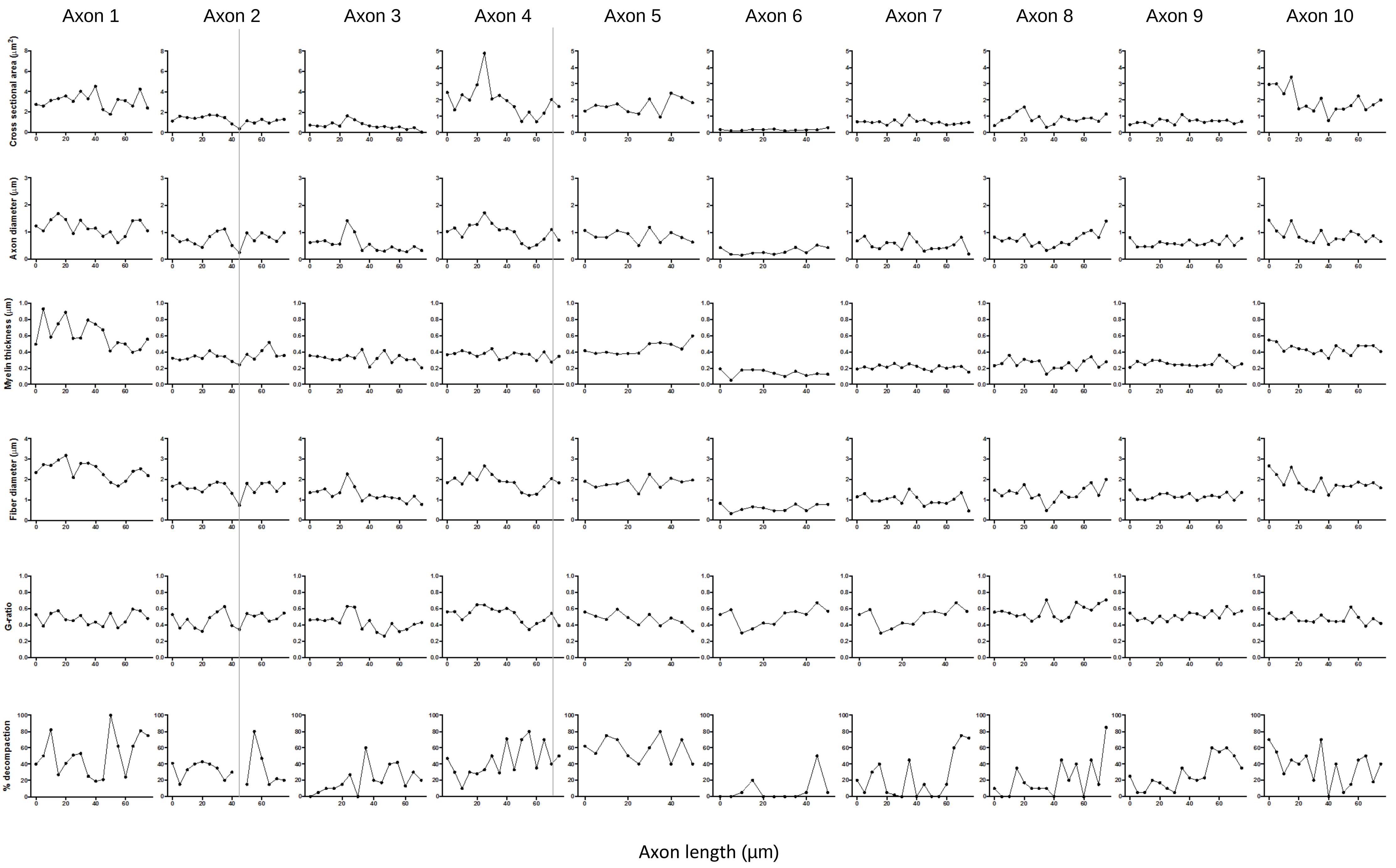
Axon 1
Axon 2
Axon 3
Axon 4
Axon 5
Axon 6
Axon 7
Axon 8
Axon 9
Axon 10
Cross sectional area (µm²)
Axon diameter (µm)
Myelin thickness (µm)
Fiber diameter (µm)
G-ratio
% decompaction
Axon length (µm)

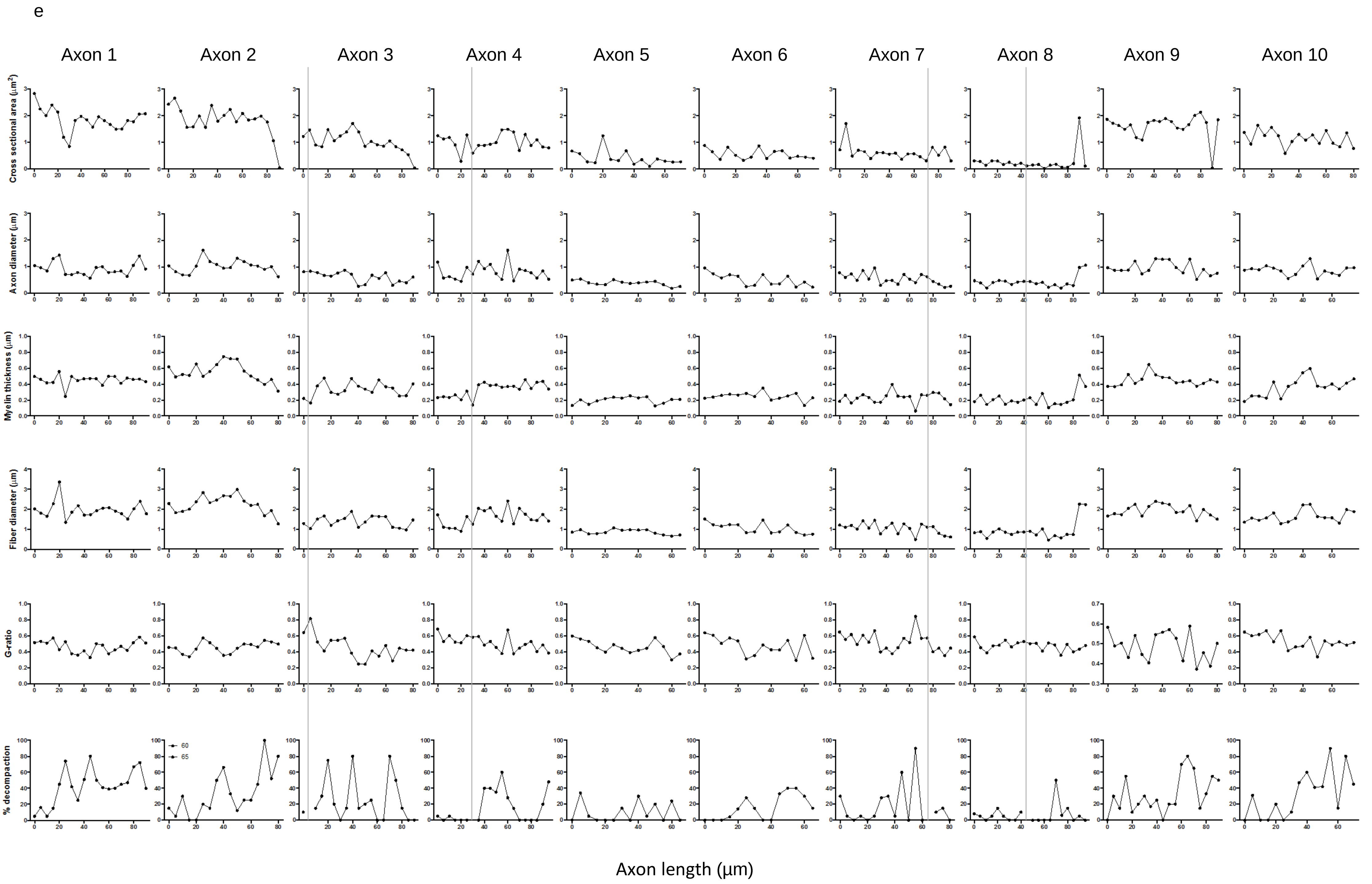
e
Axon 1
Axon 2
Axon 3
Axon 4
Axon 5
Axon 6
Axon 7
Axon 8
Axon 9
Axon 10
Cross sectional area (μm²)
Axon diameter (μm)
Myelin thickness (μm)
Fiber diameter (μm)
G-ratio
% decompaction
60
65
Axon length (μm)

Supplementary Video 1: Representative 3D rendering of the 10 randomly selected axons in optic nerve from a single normal animal shown in Fig 1, shown in video format to illustrate variability in axon dimensions along their length. L is left, R is right, S is superior, I is inferior, A is anterior, P is posterior.
